# Mir-23a induces telomere dysfunction and cellular senescence by inhibiting TRF2 expression

**DOI:** 10.1111/acel.12304

**Published:** 2015-03-06

**Authors:** Zhenhua Luo, Xuyang Feng, Haoli Wang, Weiyi Xu, Yong Zhao, Wenbin Ma, Songshan Jiang, Dan Liu, Junjiu Huang, Zhou Songyang

**Affiliations:** 1 Key Laboratory of Gene Engineering of the Ministry of Education, Key Laboratory of Reproductive Medicine of Guangdong Province, School of Life Sciences and the First Affiliated Hospital, Sun Yat-sen UniversityGuangzhou, 510275, China; 2 SYSU-BCM Joint Research Center for Biomedical Sciences and Institute of Healthy Aging Research, School of Life Sciences, Sun Yat-sen UniversityGuangzhou, 510275, China; 3 Cell-Based Assay Screening CoreOne Baylor Plaza, Houston, TX, 77030, USA; 4 Dan L. Duncan Cancer CenterOne Baylor Plaza, Houston, TX, 77030, USA; 5 Verna and Marrs McLean Department of Biochemistry and Molecular Biology, Baylor College of MedicineOne Baylor Plaza, Houston, TX, 77030, USA

**Keywords:** cellular senescence, luciferase reporter screen, miR-23a, TRF2, telomere dysfunction

## Abstract

Telomeric repeat binding factor 2 (TRF2) is essential for telomere maintenance and has been implicated in DNA damage response and aging. Telomere dysfunction induced by TRF2 inhibition can accelerate cellular senescence in human fibroblasts. While previous work has demonstrated that a variety of factors can regulate TRF2 expression transcriptionally and post-translationally, whether microRNAs (miRNAs) also participate in post-transcriptionally modulating TRF2 levels remains largely unknown. To better understand the regulatory pathways that control TRF2, we carried out a large-scale luciferase reporter screen using a miRNA expression library and identified four miRNAs that could target human TRF2 and significantly reduce the level of endogenous TRF2 proteins. In particular, our data revealed that miR-23a could directly target the 3′ untranslated region (3′UTR) of TRF2. Overexpression of miR-23a not only reduced telomere-bound TRF2 and increased telomere dysfunction-induced foci (TIFs), but also accelerated senescence of human fibroblast cells, which could be rescued by ectopically expressed TRF2. Our findings demonstrate that TRF2 is a specific target of miR-23a, and uncover a previously unknown role for miR-23a in telomere regulation and cellular senescence.

## Introduction

Telomeres are specialized protein–DNA structures at the ends of chromosomes (Blackburn, 2001; Palm & de Lange, 2008), whose dysfunction has been linked to genome instability, aging, and diseases such as cancer (Blackburn, 2001; Feldser *et al*., 2003; Martinez & Blasco, 2010; Shay & Wright, 2011; Lopez-Otin *et al*., 2013). In mammalian cells, the six-protein complex composed of TRF1, TRF2, RAP1, TIN2, TPP1, and POT1 (referred to as the telosome/shelterin) is central to ensuring telomere integrity, protecting telomeres from being recognized as DNA breaks, and coordinating telomerase-dependent maintenance of telomere length (Liu *et al*., 2004; de Lange, 2005; Palm & de Lange, 2008; Xin *et al*., 2008). Disruption of the telosome/shelterin complex and inhibition of its subunits can lead to telomere dysfunction-induced foci (TIFs), telomere recombination, chromosome end-to-end fusions, and ultimately cell senescence and death (van Steensel *et al*., 1998; Takai *et al*., 2003; Wu *et al*., 2006; Deng *et al*., 2008; Kim *et al*., 2008; Fumagalli *et al*., 2012; van Tuyn & Adams, 2012; Martinez *et al*., 2014).

Of the six core telomere proteins, telomeric repeat binding factor 2 (TRF2) is a double-stranded DNA binding protein essential for telomere end protection and T-loop formation (Griffith *et al*., 1998; van Steensel *et al*., 1998; Takai *et al*., 2003; Doksani *et al*., 2013). TRF2 also functions as a protein–protein interaction hub within the telomere interaction network, bringing together signaling molecules from numerous cellular pathways (Kim *et al*., 2009). For example, TRF2 protects telomeres from being recognized as double-strand breaks (DSBs) by repressing ataxia-telangiectasia-mutated (ATM) signaling (Karlseder *et al*., 1999; Denchi & de Lange, 2007). RNAi-mediated knockdown of TRF2 or overexpression of dominant negative TRF2 (e.g., TRF2ΔBΔM) resulted in DNA damage response at telomeres and induced apoptosis or senescence in different cell types (Karlseder *et al*., 1999; Takai *et al*., 2003, 2010). Recent studies suggest that TRF2 expression is tightly regulated by several factors. For instance, transcription factor Sp1 and β-catenin were shown to transcriptionally activate TRF2 expression (Dong *et al*., 2009; Diala *et al*., 2013). Moreover, TRF2 proteins can be degraded by the p53-induced E3 ligase Siah1 during replicative senescence in normal diploid human fibroblasts (Fujita *et al*., 2010).

Despite recent advances in our understanding of TRF2, a comprehensive picture of the regulatory pathways that control TRF2 expression and function remains elusive. For example, while accumulating evidence suggests that microRNAs (miRNAs) constitute key regulators in a diverse array of biological processes including aging (Gorospe & Abdelmohsen, 2011), whether and how miRNAs control the expression of core telomere proteins is largely unknown. miRNAs are a group of endogenous small noncoding RNAs (usually 21–24 n.t.) that regulate gene expression mainly through targeting the 3′ untranslated regions (3′UTRs) of mRNAs (Ambros, 2004; Bartel, 2009). It was demonstrated that miR-1207-5p and miR-1266 could suppress gastric cancer growth and invasion by targeting the telomerase reverse transcriptase (hTERT) (Chen *et al*., 2014). And miR-155, a well-known onco-miRNA, was able to target TRF1 and drive telomere fragility in breast cancer (Dinami *et al*., 2014). These recent studies have pointed to potential cross talk between miRNAs and telomere regulation.

We hypothesized that miRNAs might also be important regulators of TRF2 and developed a high-throughput luciferase reporter platform to identify candidate miRNAs that could target TRF2. Our screening of a human miRNA expression library with 553 miRNAs revealed miR-23a as a novel regulator of TRF2 expression and telomere maintenance. We further demonstrated that miR-23a could directly target TRF2 3′UTR and inhibit TRF2 expression, inducing telomere dysfunction and cellular senescence in human fibroblast cells. These findings uncover a novel function for miR-23a and highlight new regulatory mechanisms that control telomere DNA damage response and cellular senescence.

## Results

### A dual luciferase reporter screen identified miRNAs that could target TRF2 3′UTR

**Fig 1:**
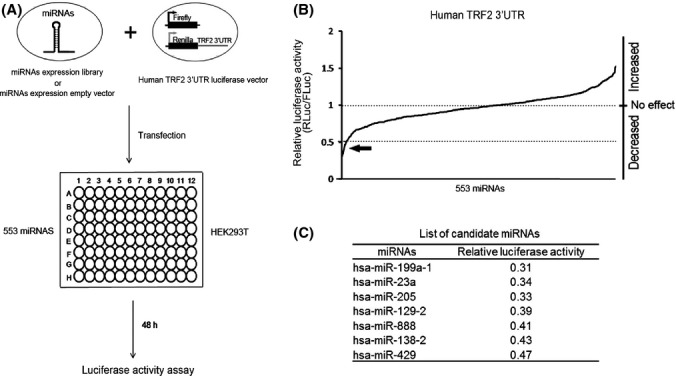
An arrayed dual luciferase reporter screen using the miRNA expression library revealed multiple miRNAs that could regulate TRF2 expression. (A) The dual luciferase reporter screen strategy. The human miRNA expression library that was arrayed in 96-well plates was co-transfected with the human TRF2–3′UTR dual luciferase reporter into HEK293T cells. All transfections were performed in triplicate. Cells were harvested 48 h after transfection for luciferase assays. An empty miRNA expression vector was also included in each plate (in triplicate) as a negative control. Potential candidates were then further examined in secondary screens. (B) Relative luciferase activity (*Renilla*/*Firefly* luciferase activity) was calculated for each miRNA and plotted. Arrowhead indicates miRNAs with relative luciferase ratios 50% lower than controls. These miRNAs are listed in (C).

To identify miRNAs that could target the 3′UTR of TRF2, we employed a dual luciferase reporter strategy (Wu *et al*., 2010; O'Loghlen *et al*., 2012; Zhang *et al*., 2012) and the human miRNA expression library (553 miRNAs) that we described previously (Zhou *et al*., 2013) (Fig.1A). Specifically, we cloned the entire 3′UTR region (1647–2981 bp of the human TRF2 locus (NCBI Reference Sequence: NM_005652.4) into the psiCHECK-2 vector just downstream of the *Renilla* luciferase (Rluc) cDNA sequence (Fig.1A)). In this system, if a miRNA could target the TRF2 3′UTR, it would impact the expression of Rluc, leading to changes in its luminescence signals. The firefly luciferase (Fluc) gene on the same vector served as an internal control for transfection efficiency.

We then carried out an arrayed dual luciferase reporter screen by co-transfecting each miRNA expression vector with the TRF2 3′UTR luciferase reporter in 96-well plates. All experiments were carried out in triplicate, and an empty miRNA expression vector was also included as a negative control in each plate. Luciferase activities were then assessed, and the ratio of Rluc/Fluc activities was calculated for each miRNA (and normalized to the empty vector control) (Supplemental Table S1). As shown in Fig.1B, the effects of these miRNAs exhibited a normal distribution, with the majority of miRNAs (∼76%) having little or no effect (< 25% change in relative luciferase activities), while the remaining miRNAs had either a positive or negative impact. We were primarily interested in miRNAs that could inhibit TRF2 expression and therefore focused on miRNAs that could decrease the Rluc/Fluc activity ratio by > 50% (Fig.1C).

### Multiple miRNAs could suppress TRF2 mRNA and protein levels

**Fig 2:**
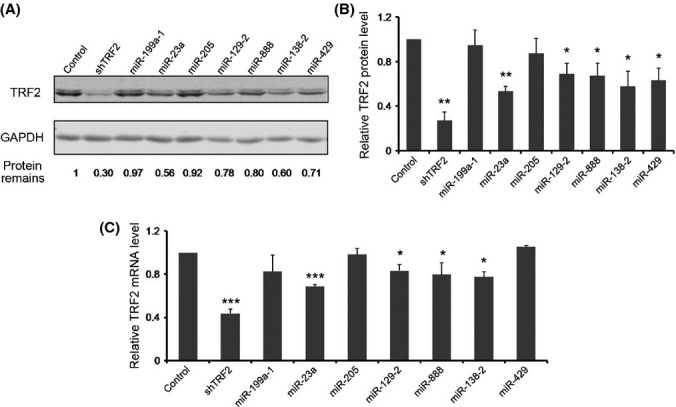
Multiple miRNAs are capable of downregulating expression of endogenous TRF2. HEK293T cells transiently expressing the miRNAs identified from our screen were analyzed. The miRNA empty vector and an shRNA vector against TRF2 (shTRF2) served as negative and positive controls, respectively. Three independent Western blotting experiments were performed using these cells. A representative image is shown in (A), with GAPDH serving as loading control. Quantification of Western blotting data is shown in (B), where error bars represent standard deviation (*n *=* *3). *P* values were determined by Student's *t*-test. *P <* *0.05 and **P <* *0.01. (C) The cells were also examined by qPCR. And GAPDH served as the internal control. Error bars represent standard deviation (*n *=* *3). *P* values were determined by Student's *t*-test. *P <* *0.05; **P <* *0.01; and ***P <* *0.001.

We next investigated whether the candidate miRNAs from our dual luciferase reporter screen (Fig.1C) could inhibit endogenous TRF2 expression. The seven miRNAs were individually transfected into HEK293T cells for subsequent Western blotting and quantitative RT–PCR analysis to determine endogenous TRF2 protein and mRNA levels. As a positive control for TRF2 inhibition, we used an shRNA vector against TRF2 (shTRF2), which could reduce TRF2 protein and mRNA levels by ∼80% (Fig.2A). Of the candidate miRNAs we examined, miR-23a, miR-129-2, miR-888, and miR-138-2 could suppress both mRNA and protein levels, with miR-23a being the most effective (Fig.2B,C). These findings indicated that our dual luciferase screen was able to identify miRNAs that downregulated endogenous TRF2 expression and that miR-23a may be a novel regulator of telomeres by directly targeting TRF2.

### miR-23a can directly target TRF2 3′UTR

**Fig 3:**
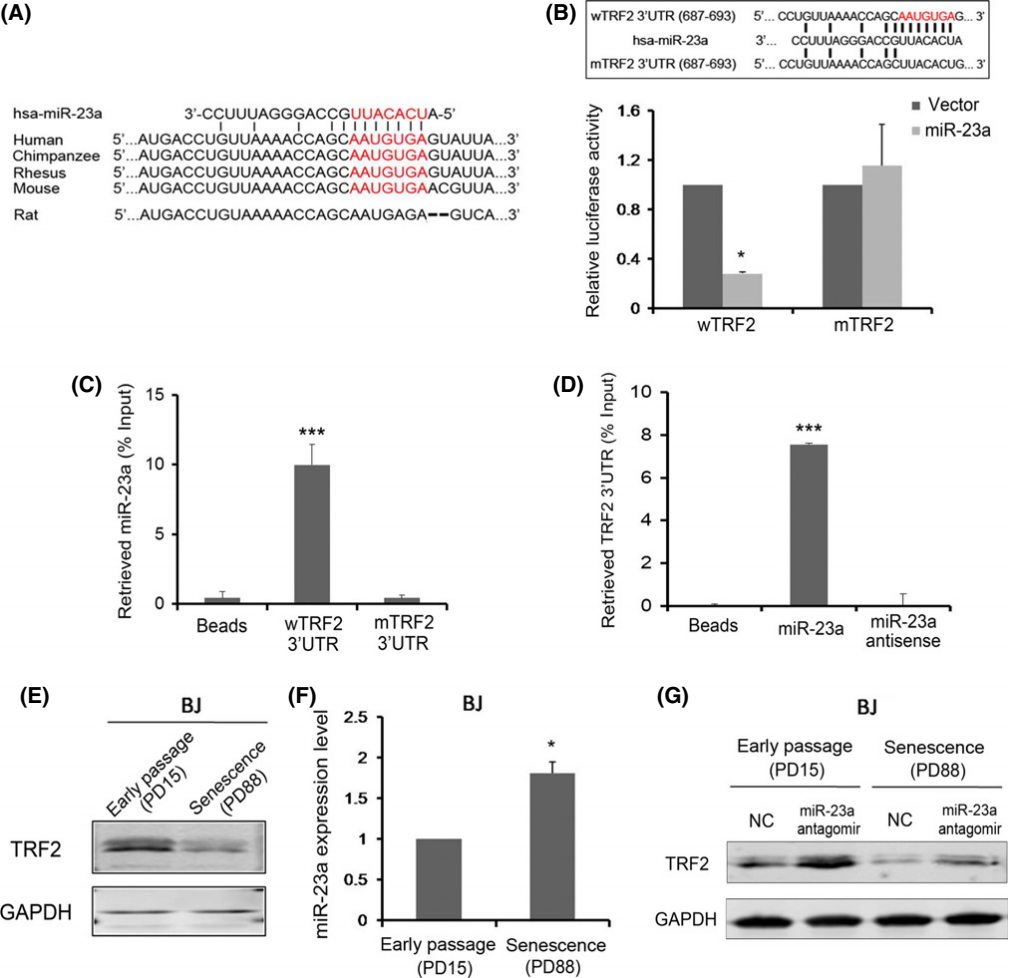
MiR-23a can directly target TRF2 3′UTR. (A) The predicted target site of miR-23a within TRF2 3′UTR appears conserved across different species. The putative seed region is highlighted in red. (B) Point mutations within the seed region (red) of the predicted TRF2 3′UTR target site were generated. The ability of miR-23a to target wild-type (wTRF2) vs. mutant (mTRF2) TRF2 3′UTR was compared in dual luciferase reporter assays. Error bars represent standard deviation (*n *=* *3). *P* values were determined by Student's *t*-test. *P <* *0.05. (C) Lysates from BJ fibroblast cells were incubated with biotin-labeled oligos of full-length wild-type and mutant TRF2 3′UTR for streptavidin pull-down and qPCR detection of miR-23a. Error bars represent standard deviation (*n *=* *3). *P* values were determined by Student's *t*-test. ***P <* *0.001. (D) Lysates from BJ fibroblast cells were incubated with biotin-labeled miR-23a wild-type or antisense oligos. TRF2 3′UTR was detected by qPCR following streptavidin pull-down. Error bars represent standard deviation (*n *=* *3). *P* values were determined by Student's *t*-test. ***P <* *0.001. (E) Early passage (PD15) and replicative senescent (PD88) BJ fibroblasts were harvested and examined by Western blotting using the indicated antibodies. GAPDH serves as loading control. (F) Cells from (E) were harvested and examined by qPCR analysis of endogenous miR-23a mRNA level. Error bars represent standard deviation (*n *=* *3). *P* values were determined by Student's *t*-test. *P <* *0.05. (G) Cells from (E) were transfected with negative control (NC) oligos or antisense oligos for miR-23a (miR-23a antagomir) and harvested for Western blotting. GAPDH serves as loading control.

Using five different miRNA target prediction programs, TargetScan, DIANAmT, miRanda, miRWalk, and RNA22, we derived a putative miR-23a target site within the TRF2 3′UTR (687–693 bp) that appears conserved among primates and mice (Fig.3A).

To determine whether the putative site could indeed be targeted by miR-23a, we mutated the predicted seed region (2-8nt) (Bartel, 2009) and generated a TRF2 3′UTR mutant (Fig.3A,B). Using dual luciferase reporter assays, we compared the effect of miR-23a on Rluc/Fluc activity ratios when tested against wild-type vs. mutant 3′UTRs and found that the relative luciferase activities were reduced by ∼70% with wild-type 3′UTR (wTRF2) but remained unchanged with the 3′UTR mutant (mTRF2) (Fig.3B), indicating that the mutated region was indeed important for miR-23a-mediated regulation of TRF2 expression. Furthermore, we were able to pull down endogenous miRNA23a using biotin-labeled TRF2 3′UTR, and vice versa with labeled miR-23a (Fig.3C,D). These data indicate specific association between miR-23a and TRF2 3′UTR and support the notion that miR-23a could directly target TRF2 by base-pairing with its target site within TRF2 3′UTR.

It was shown previously that TRF2 could function downstream of p53 and that TRF2 expression was reduced during replicative senescence in normal human diploid fibroblasts (Fujita *et al*., 2010). When we examined TRF2 expression in human BJ and MRC-5 fibroblast cells, we also found significantly downregulated TRF2 protein expression when these cells became senescent (PD88) (Fig.3E and Supplemental Figure S1A). Interestingly, we found upregulated expression of miR-23 (∼twofold) to coincide with the reduction in TRF2 levels in replicative senescent BJ (PD88) and MRC-5 cells (PD58) (Fig.3F and Supplemental Figure S1B). To further confirm the effect of miR-23a on TRF2 expression regulation, we introduced into early passage and senescent cells antisense oligos against endogenous miR-23a (miR-23a antagomir). Consistent with the ability of miR-23a to negatively regulate TRF2 expression, we observed increased levels of endogenous TRF2 in these cells (Fig.3G). Taken together, these data support our hypothesis that miR-23a could negatively regulate TRF2 expression during replicative senescence.

### Overexpression of miR-23a led to telomere deprotection by reducing telomere-bound TRF2 proteins

**Fig 4:**
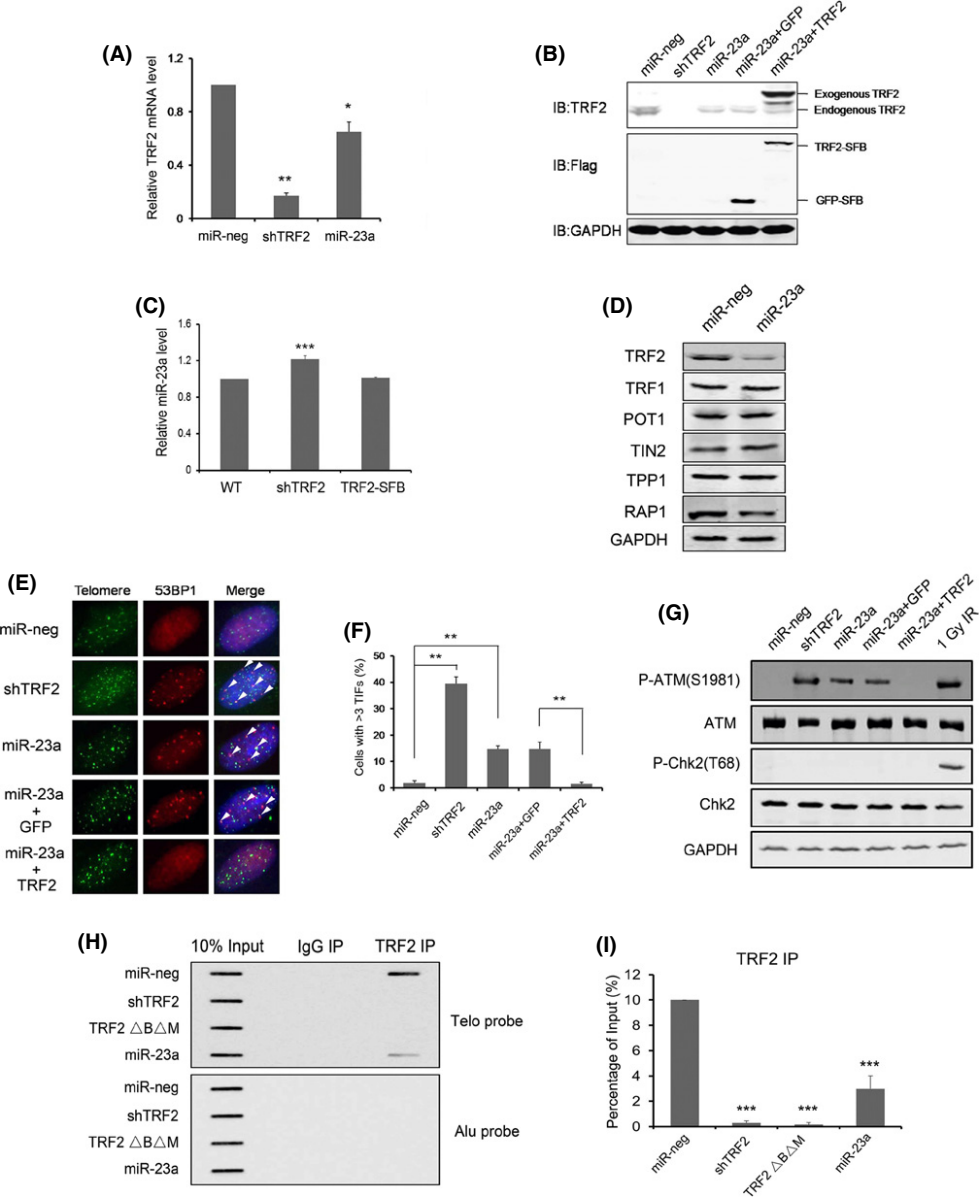
MiR-23a overexpression leads to increased telomere dysfunction-induced foci (TIF) and reduced targeting of TRF2 to telomeres. (A) Early passage BJ fibroblasts stably expressing miR-neg (miRNA negative control), shTRF2, or miR-23a were examined by qPCR. Error bars represent standard deviation (*n *=* *3). *P* values were determined by Student's *t*-test. *P <* *0.05 and **P <* *0.01. (B) Early passage BJ fibroblasts stably expressing miR-neg, shTRF2, miR-23a alone, or miR-23a plus SFB-tagged GFP or TRF2 were harvested for Western blotting analysis using the indicated antibodies. GAPDH serves as loading control. (C) Parental BJ fibroblast cells (WT) and those stably expressing TRF2-SFB or shTRF2 were examined by qPCR to detect miR-23a levels. U6 served as the internal control. Error bars represent standard deviation (*n* = 3). *P* values were determined by Student's *t*-test. **P < 0.01. (D) Cells overexpressing miR-neg or miR-23a were harvested and examined by Western blotting using the indicated antibodies, with GAPDH serving as loading control. (E) Cells from (B) were examined by immunostaining using an antibody against 53BP1 (red) and the PNA-TeloC-FITC probe (green). Nuclei were stained with DAPI. Only cells with > 3 co-localized 53BP1/telomere foci were scored as TIF positive, and > 100 cells were examined for each sample. Three independent experiments were performed. Arrowheads indicate co-localized foci. (F) Quantification of TIF data from (E). Error bars represent standard deviation (*n *=* *3). *P* values were determined by Student's *t*-test. **P <* *0.01. (G) Cells from (B) were harvested and examined by Western blotting using the indicated antibodies. Cells treated with low-dose IR (1 Gy) were used as positive controls. (H) Cells from (A) were harvested and examined by Telomere ChIP assays. Cells stably expressing the TRF2 truncation mutant TRF2ΔBΔM were used as positive controls. The precipitated DNA was slot-blotted and probed with a telomere probe. IgG served as immunoprecipitation (IP) control, and the Alu probe was used for loading control. Three independent experiments were carried out. A representative blot is shown here. (I) quantification of the ChIP data from (H). TRF2 ChIP data were normalized to control IgG IPs and plotted. Error bars represent standard deviation (*n *=* *3). *P* values were determined by Student's *t*-test. ***P <* *0.001.

**Fig 5:**
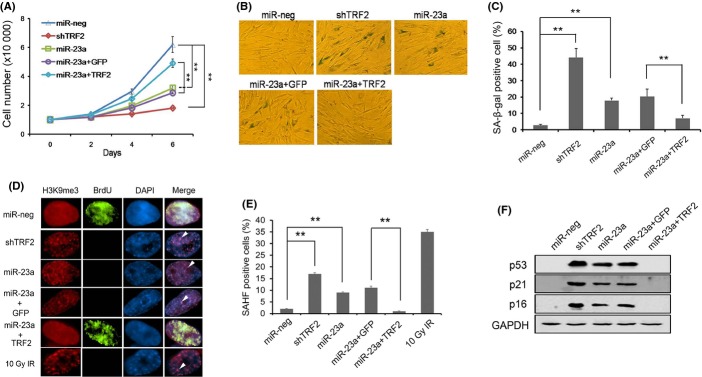
MiR-23a-induced senescence phenotypes could be rescued by TRF2. (A) Early passage BJ cells were transduced with viruses encoding empty vector (miR-neg), shTRF2, miR-23a, mir-23a plus GFP, or miR-23a plus TRF2, selected, and then monitored for growth at different time points following drug selection. Three independent experiments were performed. Error bars represent standard deviation (*n *=* *3). *P* values were determined by Student's *t*-test. **P <* *0.01. (B) Cells from (A) were stained for β-galactosidase activity (SA-β-gal). Three independent experiments were performed. (C) Quantification of the SA-β-gal data from (B). Error bars represent standard deviation (*n *=* *3). *P* values were determined by Student's *t*-test. **P <* *0.01. (D) Cells from (A) were stained for senescence-associated heterochromatin formation (SAHF). Antibodies against H3K9me3 (red) and BrdU (green) were used, and nuclei were stained with DAPI. Arrowheads indicate co-localized and condensed foci. Three independent experiments were performed. (E) Quantification of SAHF data from (D). More than 100 cells were scored per experiment. Error bars represent standard deviation (*n *=* *3). *P* values were determined by Student's *t*-test. **P <* *0.01. (F) Cells from (A) were harvested and examined by Western blotting using the indicated antibodies. GAPDH serves as loading control.

Given the importance of TRF2 in telomere protection, we postulated that increased expression of miR-23a should inhibit TRF2 function and disrupt normal telomere maintenance. To test this possibility, we stably expressed miR-23a in early passage BJ fibroblasts. The shRNA vector against TRF2 (shTRF2) and a nontargeting miRNA sequence (miR-neg) served as positive and negative controls, respectively. The same multiplicity of infection (MOI) was used for all transductions to eliminate variations in plating efficiency. As expected, shTRF2 significantly knocked down the expression of endogenous TRF2 mRNA (∼80%) and protein (∼95%) levels as determined by quantitative RT–PCR and Western blotting (Fig.4A,B). In comparison, ectopic expression of miR-23a led to ∼35% and ∼80% decrease, respectively, in TRF2 mRNA and protein levels (Fig.4A,B), indicating that increased miR-23a expression likely resulted in TRF2 mRNA degradation and translational inhibition. miR-23a targeting of TRF2 appeared to be specific, because the protein levels of other telosome/shelterin components, such as TRF1, POT1, TIN2, and TPP1, were unaffected (Fig.4D). As TRF2-RAP1 interaction is critical for RAP1 protein stability (Celli & de Lange, 2005), we also noticed that endogenous RAP1 level decreased in miR-23a overexpressed cells (Fig.4D). To determine whether TRF2 could also affect miR-23a expression, we compared miR-23a levels in early passage (PD18) parental BJ fibroblasts with cells ectopically expressing full-length TRF2 or TRF2 shRNA. Of the three cell lines, we only observed a slight increase (∼20%) in miR-23a expression in TRF2 knockdown cells (Fig.4C), perhaps a result of increased p53 levels in TRF2 knockdown cells (Fig.5F).

We next investigated whether the changes in TRF2 expression were accompanied by telomere deprotection and examined the formation of telomere dysfunction-induced foci (TIFs) in BJ cells stably expressing miR-23a. Co-staining of DNA damage response protein 53BP1 and telomeres (TIFs) would indicate damage at telomeres. As shown in Fig.4E, F, overexpression of miR-23a led to a sevenfold increase in TIF-positive cells (15% vs. 2%). When we co-expressed TRF2 in these cells (Fig.4B), the percentage of TIF-positive cells decreased to a level similar to control cells (Fig.4F), whereas co-expression with GFP had no effect. These observations indicate that TIF induction as a result of miR-23a overexpression was TRF2 dependent.

As TRF2 knockdown activates ATM signaling and induces intermediate state telomeres in human primary fibroblast (Karlseder *et al*., 1999; Cesare *et al*., 2013), we next examined ATM activation in miR-23a overexpressed cells. Cells treated with low-dose ionizing radiation (IR) were used as positive controls for the experiment. As shown in Fig.4G, overexpression of miR-23a resulted in phosphorylation of ATM S1981, but not the ATM downstream effector Chk2 (T68 phosphorylation) (Fig.4G). This finding is consistent with recent findings of distinct DNA damage responses as a result of TRF2 depletion (Cesare *et al*., 2013).

We reasoned that reduced TRF2 protein levels because of miR-23a overexpression might lead to decreased targeting of TRF2 to telomere chromatin, thus resulting in TIF induction and ATM activation. We therefore performed telomere chromatin immunoprecipitation (ChIP) assays to determine the level of TRF2 proteins on the telomere chromatin. Cells expressing the TRF2 truncation mutant TRF2ΔBΔM were used as positive controls. Consistent with our hypothesis, the amount of telomere-bound TRF2 in cells overexpressing miR-23a was ∼30% of that in control cells (Fig.4H,I), adding further support to the notion that miR-23a can induce telomere dysfunction by reducing the amount of telomere-bound TRF2.

### miR-23a overexpression induced cellular senescence

TRF2 reduction and telomere dysfunction can promote cellular senescence in human fibroblast cells (Takai *et al*., 2010). To further determine the role of miR-23a in cellular senescence, we monitored the proliferation of cells ectopically expressing miR-23a. As shown in Fig.5A, forced expression of miR-23a led to decreased cell proliferation (∼twofold decrease by day 6) and enhanced activities of β-galactosidase (SA-β-gal), a senescence biomarker. Here, miR-23a significantly increased the percentage of SA-β-gal-positive cells over controls (∼6.8-fold by day 8) (Fig.5B,C and supplementary Figure S2). This is further corroborated by our immunostaining assay for senescence-associated heterochromatin formation (SAHF), another marker often found in senescent fibroblasts (Narita *et al*., 2003). Consistent with our SA-β-gal staining results, overexpression of miR-23a also elevated SAHF in these cells, with ∼10% SAHF-positive cells by day 8 compared to ∼1% in control cells (Fig.5D,E). Importantly, the above senescent phenotypes in miR-23a overexpression cells could be rescued with the co-expression of exogenous TRF2 (Fig.5A–E), further supporting the notion that miR-23a regulates telomere maintenance and senescence through direct inhibition of TRF2. Furthermore, we also observed accumulation of p53, p21, and p16 in miR-23a overexpressing cells, implying that miR-23a induced cell growth inhibition and senescence was p53 dependent (Fig.5F).

## Discussion

In this study, we employed a dual luciferase reporter screening approach to identify miRNAs that could target the human telomeric protein TRF2. Similar strategies have been used to uncover miRNAs that can target p21, Cbx7, and Smad4 (Wu *et al*., 2010; O'Loghlen *et al*., 2012; Zhang *et al*., 2012). Our successful identification of regulatory miRNAs for TRF2 not only highlights the power of this luciferase reporter approach to discover functional miRNAs, but also underlines the fact that individual genes may be regulated by multiple miRNAs (Zhou *et al*., 2013). Here, we identified four miRNAs, including miR-23a, that could repress endogenous TRF2 mRNA and protein expression, expanding the regulatory network that modulates TRF2 and telomere function.

Multiple lines of evidence support direct regulation of TRF2 by miR-23a. miR-23a was previously reported to be upregulated in replicative senescent human umbilical vein endothelial cells and human umbilical cord blood-derived multipotent stem cells (Lee *et al*., 2011; Dellago *et al*., 2013). In senescent BJ and MC-5 human fibroblast cells, we also observed upregulated miR-23a levels; additionally, a concomitant reduction of TRF2 expression in both mRNA and protein levels was also apparent. Importantly, when we mutated the highly conserved putative miR-23a target site within TRF2 3′UTR, it abrogated the ability of miR-23a to negatively regulate TRF2 expression.

Data from previous studies support the importance of miR-23a in controlling cell growth and proliferation (Chhabra *et al*., 2009). In mammalian cells, TRF2 inhibition uncaps telomeres, leading to DNA damage response and senescence (Takai *et al*., 2003, 2010; Miranda *et al*., 2006). We have provided evidence here that miR-23a could regulate TRF2-dependent telomere maintenance and senescence control; more importantly, ectopic expression of TRF2 could rescue the phenotypes caused by miR-23a overexpression. These data suggest that miR-23a may function as a general senescence regulatory miRNA in mammalian cells and that TRF2 is the main target in miR23a-mediated senescence pathways. p53 was shown to activate the E3 ligase Siah1, which in turn ubiquitinated and degraded TRF2 in senescent human fibroblasts (Fujita *et al*., 2010). In addition, activation of p53 by nutlin-3α treatment could upregulate miR-23a level in hepatocellular carcinoma cell lines (Wang *et al*., 2013). Taken together with our results, these data point to a putative p53-miR-23a-TRF2 signaling cascade. Further work will be needed to better elucidate the relationship between p53 activation and miR-23a activity.

The proper expression level of TRF2 is essential for telomere capping, and DNA damage and repair (van Steensel *et al*., 1998; Cesare *et al*., 2013). Because dramatically reducing TRF2 levels can lead to deleterious consequences such as end-to-end fusions and compromise cell survival (Palm & de Lange, 2008), it is therefore interesting to note that human and other species have evolved mechanisms such as relying on miRNAs to reduce such an important protein. It should be noted that the reduction of a target gene by any particular miRNA is often modest (Baek *et al*., 2008). In the case of miR-23a, its overexpression did not repress TRF2 expression to a level that would induce chromosome end-to-end fusions (data no shown). Perhaps by inducing telomere dysfunction and cellular senescence, miR-23a mediated regulation of TRF2 functions as a part of the p53-induced senescence mechanism to avoid cell immortality. In addition, some studies also suggest that miRNAs can serve as gene expression buffers by reducing the variation in expression levels of the target genes (Wu *et al*., 2009). Consequently, TRF2 regulation by miR-23a may provide adaptive mechanisms for cells in response to different signaling stimuli and regulatory networks. Together with recent findings of miR-155 regulation of TRF1 in breast cancer (Dinami *et al*., 2014), our findings underscore the importance of miRNAs in controlling normal cell senescence and aging processes and suggest new avenues to explore the signaling pathways that regulate telosome/shelterin-mediated telomere maintenance.

## Experimental procedures

### Vectors and sequences

Genomic fragments containing human miRNA precursors were cloned into a modified pLL3.7 vector, and construction of the human miRNA expression library has been described previously (Zhou *et al*., 2013). The MiR-neg (miRNA negative control) sequence was amplified from the pcDNA6.2-GW/±EmGFP-miR-neg plasmid (gift from Dr. Liang-hu Qu from SYSU). The entire 3′UTR of human TRF2 (1647–2981 bp of human TRF2 locus, NCBI Reference Sequence: NM_005652.4) was cloned into the psiCHECK-2 vector (Promega, Madison, WI, USA) downstream of the *Renilla* luciferase reporter gene (named TRF2-3′UTR luciferase reporter). Mutations in the seed region of miR-23a binding site in the TRF2 3′UTR were generated by overlap extension PCR. TRF2 shRNA sequence (5′-ACAGAAGCAGTGGTCGAATC-3′) was cloned to the pLKO.1 lentiviral vector (shTRF2). cDNAs encoding GFP, full-length human TRF2 without 3′UTR, or TRF2 ΔBΔM truncation mutant were tagged at the C-terminus with SFB tag (S-tag, Flag epitope tag, and streptavidin-binding peptide tag) and cloned into pBabe-based retroviral vectors as previously described (Kim *et al*., 2009).

Control miRNA antagomir negative oligo (NC) and miR-23a antagomir oligo were purchased from Genepharma; miRNA antagomir negative control (NC): 5′-UUGUACUACACAAAAGUACUG-3′ and miR-23a antagomir: 5′-GGAAAUCCCUGGCAAUGUGAU-3′.

### Cell lines, Western blotting, and antibodies

HEK293T cells and the normal human diploid fibroblast cell lines MRC-5 and BJ were cultured in DMEM/high-glucose medium (Thermo Scientific, USA) supplemented with 10% fetal bovine serum and penicillin/streptomycin (Thermo Scientific, USA). To generate stable expression BJ cells, transduction was carried out with the same multiplicity of infection (MOI) for different lentiviruses. Cells were selected with 1 μg mL^−1^ puromycin for > 48 h postinfection. For rescue experiments, miR-23a expressing cells were superinfected with retroviruses encoding GFP-SFB or TRF2-SFB and selected with 1 μg mL^−1^ puromycin 24 h later. BJ fibroblasts were transfected with 50 nm miRNA antagomir oligos using RNAiMAX (Invitrogen, Carlsbad, CA, USA, 13778).

Western blotting was carried out as previously described Wang *et al*. (2013). Samples were resolved by SDS-PAGE and transferred to nitrocellulose membranes (Millipore, Billerica, MA, USA), before blocking and incubation with antibodies. The membranes were analyzed using the Odyssey Imaging System (LI-COR, Lincoln, Nebraska USA). Antibodies used in this study were mouse monoclonal anti-TRF2 (Calbiochem, Billerica, MA, USA, OP-129), rabbit polyclonal anti-POT1 (Novus Biologicals, Littleton, CO, USA, NB500-176), rabbit anti-RAP1 (Bethyl Laboratories, Montgomery, TX, USA, A300-306A), rabbit anti-TPP1 and anti-TIN2 (Songyang lab, Guangzhou, Guangdong, China), goat anti-TRF1 (Songyang lab, Guangzhou, Guangdong, China), mouse monoclonal anti-GAPDH (Abmart, Shanghai, China, 3B3), rabbit polyclonal anti-FLAG (Sigma, F7425), mouse IgG (Sigma, St. Louis, MO, USA, I5381), rabbit polyclonal anti-H3K9me3 (Abcam, Cambridge, MA, USA, ab71999), rabbit polyclonal anti-53BP1 (Novus Biologicals, Littleton, CO, USA, NB100-904), mouse monoclonal anti-phospho-ATM (Ser1981) (Millipore, Billerica, MA, USA, 05-740), mouse monoclonal anti-ATM (Millipore, Billerica, MA, USA, 05-513), mouse monoclonal anti-Chk2 (Millipore, Billerica, MA, USA, 05-649), anti-phospho-Chk2 (Thr68) (Cell Signaling Technology, Danvers, MA, USA, 2661), mouse monoclonal anti-p53 (Santa Cruz Biotechnology, Santa Cruz, CA, USA, sc-126), mouse monoclonal anti-p21WAF1 (Millipore, Billerica, MA, USA, OP-64-100UGCN), mouse monoclonal anti-p16INK4a (BD PharMingen, San Jose, CA, USA, 554079), mouse monoclonal anti-BrdU (Sigma, St. Louis, MO, USA, B2531), IRDye-800CW goat anti-mouse (LI-COR, Lincoln, Nebraska USA, 926-32210), IRDye-800CW goat anti-rabbit (LI-COR, Lincoln, Nebraska USA, 926-32211), IRDye-800CW donkey anti-goat (LI-COR, Lincoln, Nebraska USA, 926-32214), Alexa Fluor®488 goat anti-mouse IgG (Invitrogen, Carlsbad, CA, USA, A11001), and Texas-Red goat anti-rabbit IgG (Invitrogen, Carlsbad, CA, USA, T2767).

### Dual luciferase reporter assay screen

The dual luciferase reporter assay was performed as previously described (Zhou *et al*., 2013). Briefly, HEK293T cells (2 × 10^4^) were seeded in 96-well plates roughly 20 h before being transfected with the empty vector or individual human miRNA expression vectors (0.15 μg each) together with the TRF2-3′UTR-psiCHECK-2 vector (0.05 μg), using Fugene HD (Roche, Mannheim, Germany). The library contained 553 miRNA precursors. Each transfection was performed in triplicate. Cells were harvested 48 h post-transfection with 1xPassive buffer (Promega, Madison, WI, USA). *Renilla* and *firefly* luciferase activities were measured using the Dual-Luciferase Reporter Assay System Kit (Promega, Madison, WI, USA). The ratio of Renilla to firefly luciferase activities in the miRNA library group was normalized to the average of the miRNA empty vector group that had been included in each plate. The data collected from different 96-wells plates were integrated by combination of the normalized ratios. The mean for all three triplicates was calculated for each miRNA, and standard deviation (SD) was then determined. Student's t-test was carried out to determine significant differences, where *P *<* *0.05 was considered to be statistically significant.

### RNA pull-down assay

Full-length wild-type and mutant TRF2 3′UTR that were cloned into pCDNA3 vector were *in vitro* transcribed and biotin-labeled with the Biotin RNA Labeling Mix (Roche, Mannheim, Germany) using MEGAshortscript T7 kit (Ambion, Carlsbad, CA, USA) and then purified with the RNeasy Mini Kit (Qiagen, Valencia, CA, USA). Biotin-labeled miR-23a and miR-23a antisense oligos were purchased from Genepharma; miR-23a: 5′-biotin-AUCACAUUGCCAGGGAUUUCC-3′ and miR-23a anti-sense: 5′-biotin-GGAAAUCCCUGGCAAUGUGAU-3′. Whole-cell lysates from BJ fibroblast cells (1 mg) were incubated with biotinylated transcripts (3 μg) or oligos (1OD) for 3 hr at 4 °C. The complexes were subsequently isolated with streptavidin agarose beads (Invitrogen, Carlsbad, CA, USA) for qPCR analysis.

### Quantitative RT–PCR (qRT–PCR)

Real-time qPCR for TRF2 was carried out as described previously (Wang *et al*., 2013). Briefly, total RNAs were isolated using QIAGEN RNeasy Kit (Qiagen, Valencia, CA, USA) and reverse-transcribed using the iScript cDNA Synthesis Kit (Bio-Rad, Hercules, CA, USA). GAPDH was used as internal control for normalization. For miR-23a, total RNAs were isolated using Trizol (Invitrogen, Carlsbad, CA, USA) and reverse-transcribed using ReverTra-Ace-α-Transcriptase (TOYOBO, Japan). U6 small nuclear RNAs were used as an internal control for normalization. All qPCRs were performed using SYBR green master mix (Applied Biosystems, USA) on Applied Biosystems One-step-plus system.

The primers for miR-23a and U6 were purchased from Ribobio, Guangzhou, China.

miR-23a-RT: 5′-GTCGTATCCAGTGCAGGGTCCGAGGTGCACTGGATACGACGGAAATCC-3′
miR-23a-forward: 5′-TGCGGATCACATTGCCAGG-3′
U6-RT: 5′-GTCGTATCCAGTGCAGGGTCCGAGGTGCACTGGATACGACAAAATATGG-3′ U6-forward: 5′-TGCGGTGCTCGCTTCGGCAGC-3′
U6-Reverse: 5′-CCAGTGCAGGGTCCGAGT-3′

The TRF2 primers are TRF2-forward (5′- CCCAAGAACAAGCGCATGAC-3′) and TRF2-reverse (5′-GGGTTGGTTGAGAACGGTGG-3′). The GAPDH primers are GAPDH-forward (5′-GGAGCGAGATCCCTCCAAAAT-3′) and GAPDH-reverse (5′-GGCTGTTGTCATACTTCTCATGG-3′).

The TRF2 3′UTR qPCR primers for RNA pull-down are TRF2 3′UTR-forward (5′- CCAGGTTGATGACAGACCAG-3′) and TRF2 3′UTR-reverse (5′-AGATGTTGACAGCAAATGCC-3′).

### Indirect immunofluorescence (IF) and IF-fluorescent *in situ* hybridization (IF-FISH)

Indirect immunofluorescence was carried out essentially as previously described (Wang *et al*., 2013). Cells plated on glass coverslips were fixed with 4% paraformaldehyde, permeabilized in 0.5% Triton X-100 and blocked with 5% goat serum before primary and secondary antibodies (all are listed above) incubation. For IF-FISH, an additional incubation with PNA-TelC-FITC probe (Panagene, Daejeon, Korea) was performed at 37 °C for 2 h after secondary antibody incubation. The coverslips were mounted onto glass slides with DAPI-containing VECTASHIELD Mounting medium (Vector Labs, Burlingame, CA, USA). Fluorescence microscopy was performed on a Nikon Ti microscope. For quantification, at least 100 cells were examined for each sample.

For BrdU labeling in SAHF experiment, cells were plated on coverslips and subsequently labeled with 5-Bromo-2′-deoxyuridine (BrdU, 100 μg mL^−1^, Sigma, St. Louis, MO, USA). BrdU incorporation was visualized by immunolabeling using anti-BrdU antibody (Sigma, St. Louis, MO, USA, B2531).

### Chromatin immunoprecipitation

Chromatin immunoprecipitation (ChIP) was performed as previously described (Wang *et al*., 2013). Briefly, cells were fixed with 1% formaldehyde and collected by scraping. Cell lysate was sonicated and precleared with protein A/G agarose beads (Thermo Scientific, USA). The precleared lysate was incubated with anti-TRF2 antibody or mouse IgG control. Input and eluted DNA was further purified using QIAquik PCR purification kit (Qiagen, Valencia, CA, USA), slot-blotted onto Hybond-N+ nylon membranes (GE Healthcare, Buckinghamshire, UK). The membranes were then probed with biotin-labeled probes: the Telo probe, 5′-Biotin-TTAGGGTTAGGGTTAGGGT and the Alu probe, 5′-Biotin-GGCCGGGCGCGGTGGCTCACGCCTGTAATCCCAGCA). The signals were detected using Chemiluminescent Nucleic Acid Detection Module (Thermo Scientific, USA).

### Cell proliferation and SA-β-gal assay

To quantify cell proliferation, a total of 1 × 10^4^ cells were seeded onto 6-well plates in triplicate at 48 h postselection. At different time points, cells were trypsinized and counted. Three independent experiments were performed. To examine β-gal staining, a total of 1 × 10^4^ cells were seeded onto 6-well plates. SA-β-gal staining was performed 7 days postselection using Senescence β-Galactosidase Staining Kit (Cell Signaling Technology, Beverly, MA, USA). More than 100 cells were counted for this assay.

## Supporting Information

**Fig. S1** TRF2 and miR-23a expression in MRC-5 fibroblast cells. **Fig. S2** MiR-23a induced positive senescence β-Galactosidase staining (SA-β-gal) in MRC-5 fibroblast cells. **Table S1** Complete list of results from our dual luciferase reporter screen.
