# Supplementary material for: Mir-23a induces telomere dysfunction and cellular senescence by inhibiting TRF2 expression
Source: Aging Cell. 2015 Mar 6;14(3):391–9. doi: 10.1111/acel.12304 (PMC4406668; doi:10.1111/acel.12304)
Supplement: Supplementary file 1 [file acel0014-0391-sd1.pdf]

Luo et al. Figure S1

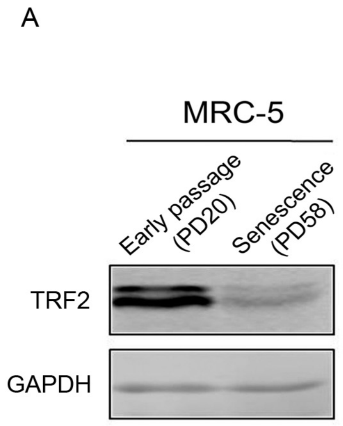

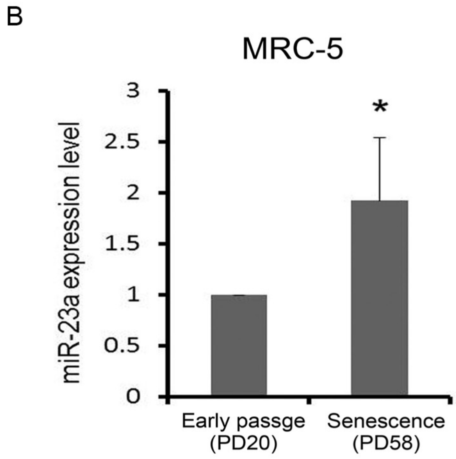

Luo et al. Figure S2

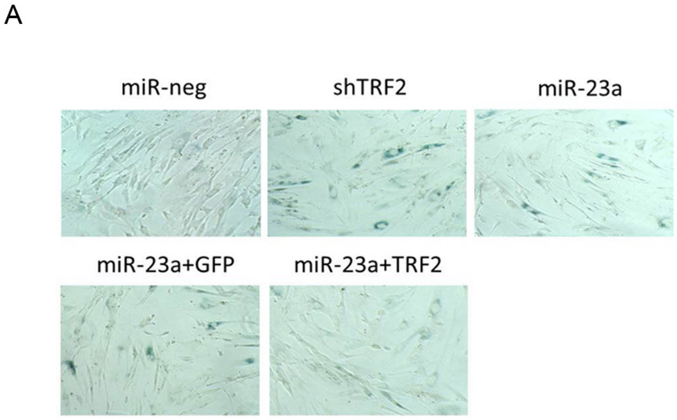

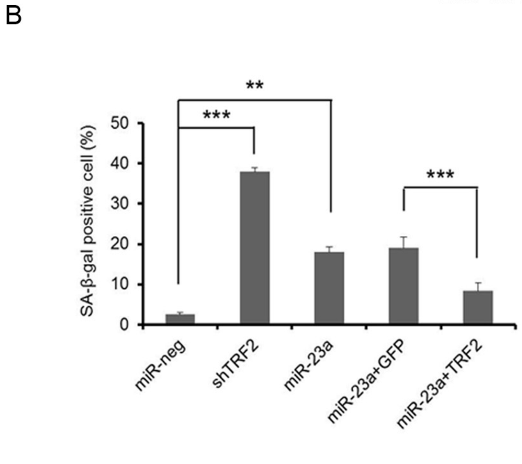

Supplemental Table S1 Luciferase reporter screen result.

miRNAs	Target	Relative luciferase activity	P value
hsa-mir-199a-1	TRF2 3'UTR	0.31	0.0024
hsa-mir-205	TRF2 3'UTR	0.33	0.0005
hsa-mir-23a	TRF2 3'UTR	0.34	0.0133
hsa-mir-129-2	TRF2 3'UTR	0.39	0.0334
hsa-mir-888	TRF2 3'UTR	0.41	0.0034
hsa-mir-138-2	TRF2 3'UTR	0.43	0.005
hsa-mir-429	TRF2 3'UTR	0.47	0.0021
hsa-mir-107	TRF2 3'UTR	0.5	0.0026
hsa-mir-1278	TRF2 3'UTR	0.5	0.0264
hsa-mir-544	TRF2 3'UTR	0.5	0.0022
hsa-mir-543	TRF2 3'UTR	0.51	0.0004
hsa-mir-615	TRF2 3'UTR	0.52	0.0041
hsa-mir-193a	TRF2 3'UTR	0.53	0.0189
hsa-mir-224	TRF2 3'UTR	0.53	0.0066
hsa-mir-206	TRF2 3'UTR	0.54	0.0024
hsa-mir-619	TRF2 3'UTR	0.56	0.0004
hsa-mir-199a-2	TRF2 3'UTR	0.57	0.0277
hsa-mir-23b	TRF2 3'UTR	0.57	0.0024
hsa-mir-892b	TRF2 3'UTR	0.57	0.0237
hsa-mir-138-1	TRF2 3'UTR	0.59	0.0055
hsa-mir-221	TRF2 3'UTR	0.6	0.0113
hsa-mir-101-1	TRF2 3'UTR	0.61	0.0188
hsa-mir-130a	TRF2 3'UTR	0.61	0.02
hsa-mir-185	TRF2 3'UTR	0.62	0.0346
hsa-mir-1296	TRF2 3'UTR	0.63	0.0317
hsa-mir-101-2	TRF2 3'UTR	0.64	0.0052
hsa-mir-1285-1	TRF2 3'UTR	0.64	0.0097
hsa-mir-193b	TRF2 3'UTR	0.64	0.0481
hsa-mir-671	TRF2 3'UTR	0.64	0.0157
hsa-mir-1255a	TRF2 3'UTR	0.66	0.0051
hsa-mir-133a-2	TRF2 3'UTR	0.66	0.0175
hsa-mir-15a	TRF2 3'UTR	0.66	0.0035
hsa-mir-382	TRF2 3'UTR	0.66	0.0055
hsa-mir-516a-1	TRF2 3'UTR	0.66	0.0232
hsa-mir-641	TRF2 3'UTR	0.66	0.0218
hsa-mir-181d	TRF2 3'UTR	0.67	0.011
hsa-mir-508	TRF2 3'UTR	0.67	0.0426
hsa-mir-629	TRF2 3'UTR	0.67	0.051
hsa-mir-656	TRF2 3'UTR	0.67	0.0889
hsa-mir-1298	TRF2 3'UTR	0.68	0.0045
hsa-mir-1979	TRF2 3'UTR	0.68	0.0321
hsa-mir-24-2	TRF2 3'UTR	0.68	0.0119
hsa-mir-297	TRF2 3'UTR	0.68	0.0357
hsa-mir-499	TRF2 3'UTR	0.68	0.0302
hsa-mir-9-2	TRF2 3'UTR	0.68	0.0192
hsa-let-7a-3	TRF2 3'UTR	0.69	0.0137

hsa-mir-448	TRF2 3'UTR	0.69	0.0046
hsa-mir-548i-1	TRF2 3'UTR	0.69	0.0346
hsa-mir-1302-2	TRF2 3'UTR	0.7	0.07
hsa-mir-133b	TRF2 3'UTR	0.7	0.0152
hsa-mir-296	TRF2 3'UTR	0.7	0.0566
hsa-mir-488	TRF2 3'UTR	0.7	0.0828
hsa-mir-491	TRF2 3'UTR	0.7	0.073
hsa-mir-7-3	TRF2 3'UTR	0.7	0.0456
hsa-mir-16-1	TRF2 3'UTR	0.71	0.0219
hsa-mir-380	TRF2 3'UTR	0.71	0.089
hsa-mir-124-2	TRF2 3'UTR	0.72	0.0332
hsa-mir-542	TRF2 3'UTR	0.72	0.0162
hsa-mir-548i-4	TRF2 3'UTR	0.72	0.0221
hsa-mir-555	TRF2 3'UTR	0.72	0.0164
hsa-mir-1257	TRF2 3'UTR	0.73	0.0157
hsa-mir-142	TRF2 3'UTR	0.73	0.0503
hsa-mir-27a	TRF2 3'UTR	0.73	0.0156
hsa-mir-767	TRF2 3'UTR	0.73	0.0991
hsa-mir-1282	TRF2 3'UTR	0.74	0.0264
hsa-mir-200b	TRF2 3'UTR	0.74	0.0053
hsa-mir-220c	TRF2 3'UTR	0.74	0.0029
hsa-mir-302d	TRF2 3'UTR	0.74	0.0393
hsa-mir-891b	TRF2 3'UTR	0.74	0.012
hsa-mir-944	TRF2 3'UTR	0.74	0.0258
hsa-mir-1274b	TRF2 3'UTR	0.75	0.041
hsa-mir-194-2	TRF2 3'UTR	0.75	0.0039
hsa-mir-379	TRF2 3'UTR	0.75	0.0339
hsa-mir-490	TRF2 3'UTR	0.75	0.022
hsa-mir-509-2	TRF2 3'UTR	0.75	0.0284
hsa-mir-516b-2	TRF2 3'UTR	0.75	0.0577
hsa-mir-29b-2	TRF2 3'UTR	0.76	0.0019
hsa-mir-300	TRF2 3'UTR	0.76	0.0788
hsa-mir-325	TRF2 3'UTR	0.76	0.0233
hsa-mir-409	TRF2 3'UTR	0.76	0.0394
hsa-mir-512-2	TRF2 3'UTR	0.76	0.0056
hsa-mir-548a-3	TRF2 3'UTR	0.76	0.0816
hsa-mir-551a	TRF2 3'UTR	0.76	0.0076
hsa-mir-553	TRF2 3'UTR	0.76	0.0678
hsa-mir-659	TRF2 3'UTR	0.76	0.1031
hsa-mir-744	TRF2 3'UTR	0.76	0.0675
hsa-mir-891a	TRF2 3'UTR	0.76	0.0959
hsa-mir-1237	TRF2 3'UTR	0.77	0.0425
hsa-mir-1471	TRF2 3'UTR	0.77	0.0302
hsa-mir-204	TRF2 3'UTR	0.77	0.044
hsa-mir-340	TRF2 3'UTR	0.77	0.0411
hsa-mir-424	TRF2 3'UTR	0.77	0.0487
hsa-mir-563	TRF2 3'UTR	0.77	0.0742
hsa-mir-1183	TRF2 3'UTR	0.78	0.1601

hsa-mir-346	TRF2 3'UTR	0.78	0.0394
hsa-mir-378	TRF2 3'UTR	0.78	0.0665
hsa-mir-507	TRF2 3'UTR	0.78	0.1461
hsa-mir-513b	TRF2 3'UTR	0.78	0.06
hsa-mir-934	TRF2 3'UTR	0.78	0.0198
hsa-mir-1185-1	TRF2 3'UTR	0.79	0.0129
hsa-mir-1254	TRF2 3'UTR	0.79	0.0289
hsa-mir-331	TRF2 3'UTR	0.79	0.029
hsa-mir-335	TRF2 3'UTR	0.79	0.0376
hsa-mir-450a-1	TRF2 3'UTR	0.79	0.0467
hsa-mir-513a-2	TRF2 3'UTR	0.79	0.0222
hsa-mir-526b	TRF2 3'UTR	0.79	0.0856
hsa-mir-1-1	TRF2 3'UTR	0.8	0.0504
hsa-mir-1-2	TRF2 3'UTR	0.8	0.0474
hsa-mir-1236	TRF2 3'UTR	0.8	0.027
hsa-mir-128-1	TRF2 3'UTR	0.8	0.0815
hsa-mir-1293	TRF2 3'UTR	0.8	0.0259
hsa-mir-223	TRF2 3'UTR	0.8	0.0498
hsa-mir-298	TRF2 3'UTR	0.8	0.0076
hsa-mir-487a	TRF2 3'UTR	0.8	0.072
hsa-mir-570	TRF2 3'UTR	0.8	0
hsa-mir-93	TRF2 3'UTR	0.8	0.0393
hsa-mir-152	TRF2 3'UTR	0.81	0.1801
hsa-mir-1537	TRF2 3'UTR	0.81	0.0115
hsa-mir-24-1	TRF2 3'UTR	0.81	0.1602
hsa-mir-25	TRF2 3'UTR	0.81	0.0326
hsa-mir-522	TRF2 3'UTR	0.81	0.0921
hsa-mir-137	TRF2 3'UTR	0.82	0.0229
hsa-mir-144	TRF2 3'UTR	0.82	0.0436
hsa-mir-146b	TRF2 3'UTR	0.82	0.1126
hsa-mir-192	TRF2 3'UTR	0.82	0.1226
hsa-mir-610	TRF2 3'UTR	0.82	0.0941
hsa-mir-631	TRF2 3'UTR	0.82	0.0227
hsa-mir-634	TRF2 3'UTR	0.82	0.0955
hsa-mir-760	TRF2 3'UTR	0.82	0.0593
hsa-let-7f-1	TRF2 3'UTR	0.83	0.1037
hsa-mir-1286	TRF2 3'UTR	0.83	0.065
hsa-mir-1299	TRF2 3'UTR	0.83	0.0206
hsa-mir-135b	TRF2 3'UTR	0.83	0.0597
hsa-mir-140	TRF2 3'UTR	0.83	0.044
hsa-mir-219-2	TRF2 3'UTR	0.83	0.0325
hsa-mir-99a	TRF2 3'UTR	0.83	0.0958
hsa-mir-1246	TRF2 3'UTR	0.84	0.1687
hsa-mir-1267	TRF2 3'UTR	0.84	0.0678
hsa-mir-1287	TRF2 3'UTR	0.84	0.0878
hsa-mir-1976	TRF2 3'UTR	0.84	0.0217
hsa-mir-1978	TRF2 3'UTR	0.84	0.0081
hsa-mir-22	TRF2 3'UTR	0.84	0.1727

hsa-mir-576	TRF2 3’UTR	0.84	0.0837
hsa-mir-583	TRF2 3’UTR	0.84	0.0665
hsa-mir-589	TRF2 3’UTR	0.84	0.0565
hsa-mir-630	TRF2 3’UTR	0.84	0.041
hsa-mir-665	TRF2 3’UTR	0.84	0.1659
hsa-mir-769	TRF2 3’UTR	0.84	0.023
hsa-mir-873	TRF2 3’UTR	0.84	0.0305
hsa-mir-95	TRF2 3’UTR	0.84	0.0534
hsa-mir-1207	TRF2 3’UTR	0.85	0.0481
hsa-mir-1226	TRF2 3’UTR	0.85	0.1575
hsa-mir-1250	TRF2 3’UTR	0.85	0.0628
hsa-mir-1271	TRF2 3’UTR	0.85	0.0854
hsa-mir-1308	TRF2 3’UTR	0.85	0.1182
hsa-mir-139	TRF2 3’UTR	0.85	0.0798
hsa-mir-1974	TRF2 3’UTR	0.85	0.0946
hsa-mir-548c	TRF2 3’UTR	0.85	0.0416
hsa-mir-548f-2	TRF2 3’UTR	0.85	0.033
hsa-mir-550-1	TRF2 3’UTR	0.85	0.1106
hsa-mir-640	TRF2 3’UTR	0.85	0.0931
hsa-mir-648	TRF2 3’UTR	0.85	0.0438
hsa-mir-664	TRF2 3’UTR	0.85	0.1438
hsa-mir-708	TRF2 3’UTR	0.85	0.114
hsa-mir-720	TRF2 3’UTR	0.85	0.012
hsa-mir-874	TRF2 3’UTR	0.85	0.1463
hsa-let-7e	TRF2 3’UTR	0.86	0.0636
hsa-mir-1184	TRF2 3’UTR	0.86	0.1038
hsa-mir-135a-1	TRF2 3’UTR	0.86	0.185
hsa-mir-1826	TRF2 3’UTR	0.86	0.0463
hsa-mir-1911	TRF2 3’UTR	0.86	0.1053
hsa-mir-1972	TRF2 3’UTR	0.86	0.0245
hsa-mir-2113	TRF2 3’UTR	0.86	0.0653
hsa-mir-302e	TRF2 3’UTR	0.86	0.0776
hsa-mir-33a	TRF2 3’UTR	0.86	0.0614
hsa-mir-523	TRF2 3’UTR	0.86	0.1003
hsa-mir-663b	TRF2 3’UTR	0.86	0.0905
hsa-mir-1269	TRF2 3’UTR	0.87	0.1886
hsa-mir-133a-1	TRF2 3’UTR	0.87	0.0101
hsa-mir-202	TRF2 3’UTR	0.87	0.0639
hsa-mir-302f	TRF2 3’UTR	0.87	0.1297
hsa-mir-486	TRF2 3’UTR	0.87	0.0456
hsa-mir-527	TRF2 3’UTR	0.87	0.0913
hsa-mir-612	TRF2 3’UTR	0.87	0.0598
hsa-mir-637	TRF2 3’UTR	0.87	0.2386
hsa-mir-649	TRF2 3’UTR	0.87	0.0741
hsa-mir-105-1	TRF2 3’UTR	0.88	0.1847
hsa-mir-106a	TRF2 3’UTR	0.88	0.0519
hsa-mir-1205	TRF2 3’UTR	0.88	0.1241
hsa-mir-1255b-2	TRF2 3’UTR	0.88	0.2623

hsa-mir-1258	TRF2 3'UTR	0.88	0.0124
hsa-mir-154	TRF2 3'UTR	0.88	0.0996
hsa-mir-21	TRF2 3'UTR	0.88	0.1903
hsa-mir-29a	TRF2 3'UTR	0.88	0.0693
hsa-mir-320b-1	TRF2 3'UTR	0.88	0.2468
hsa-mir-370	TRF2 3'UTR	0.88	0.2283
hsa-mir-521-1	TRF2 3'UTR	0.88	0.1008
hsa-mir-548k	TRF2 3'UTR	0.88	0.0332
hsa-mir-548m	TRF2 3'UTR	0.88	0.1873
hsa-mir-632	TRF2 3'UTR	0.88	0.1188
hsa-mir-876	TRF2 3'UTR	0.88	0.1701
hsa-mir-889	TRF2 3'UTR	0.88	0.2741
hsa-let-7i	TRF2 3'UTR	0.89	0.1418
hsa-mir-1204	TRF2 3'UTR	0.89	0.1977
hsa-mir-1302-8	TRF2 3'UTR	0.89	0.0578
hsa-mir-1908	TRF2 3'UTR	0.89	0.0314
hsa-mir-374a	TRF2 3'UTR	0.89	0.0783
hsa-mir-503	TRF2 3'UTR	0.89	0.1454
hsa-mir-582	TRF2 3'UTR	0.89	0.1221
hsa-mir-651	TRF2 3'UTR	0.89	0.1578
hsa-mir-1225	TRF2 3'UTR	0.9	0.234
hsa-mir-1245	TRF2 3'UTR	0.9	0.0009
hsa-mir-125b-2	TRF2 3'UTR	0.9	0.1892
hsa-mir-1295	TRF2 3'UTR	0.9	0.2527
hsa-mir-155	TRF2 3'UTR	0.9	0.3563
hsa-mir-326	TRF2 3'UTR	0.9	0.2607
hsa-mir-512-1	TRF2 3'UTR	0.9	0.1252
hsa-mir-541	TRF2 3'UTR	0.9	0.1937
hsa-mir-606	TRF2 3'UTR	0.9	0.1311
hsa-mir-638	TRF2 3'UTR	0.9	0.1351
hsa-mir-642	TRF2 3'UTR	0.9	0.1898
hsa-mir-653	TRF2 3'UTR	0.9	0.235
hsa-mir-764	TRF2 3'UTR	0.9	0.1616
hsa-mir-1231	TRF2 3'UTR	0.91	0.2524
hsa-mir-125b-1	TRF2 3'UTR	0.91	0.1452
hsa-mir-1277	TRF2 3'UTR	0.91	0.1274
hsa-mir-217	TRF2 3'UTR	0.91	0.0267
hsa-mir-301a	TRF2 3'UTR	0.91	0.1412
hsa-mir-506	TRF2 3'UTR	0.91	0.326
hsa-mir-539	TRF2 3'UTR	0.91	0.241
hsa-mir-635	TRF2 3'UTR	0.91	0.0614
hsa-mir-646	TRF2 3'UTR	0.91	0.083
hsa-mir-890	TRF2 3'UTR	0.91	0.2075
hsa-mir-937	TRF2 3'UTR	0.91	0.2422
hsa-mir-1909	TRF2 3'UTR	0.92	0.0567
hsa-mir-1912	TRF2 3'UTR	0.92	0.26
hsa-mir-1973	TRF2 3'UTR	0.92	0.2682
hsa-mir-324	TRF2 3'UTR	0.92	0.2315

hsa-mir-433	TRF2 3'UTR	0.92	0.0699
hsa-mir-628	TRF2 3'UTR	0.92	0.3784
hsa-mir-1229	TRF2 3'UTR	0.93	0.217
hsa-mir-1289-2	TRF2 3'UTR	0.93	0.2602
hsa-mir-150	TRF2 3'UTR	0.93	0.3087
hsa-mir-208b	TRF2 3'UTR	0.93	0.2553
hsa-mir-211	TRF2 3'UTR	0.93	0.195
hsa-mir-214	TRF2 3'UTR	0.93	0.3225
hsa-mir-519e	TRF2 3'UTR	0.93	0.3732
hsa-mir-551b	TRF2 3'UTR	0.93	0.2969
hsa-mir-564	TRF2 3'UTR	0.93	0.2925
hsa-mir-103-1	TRF2 3'UTR	0.94	0.2693
hsa-mir-1178	TRF2 3'UTR	0.94	0.1687
hsa-mir-1252	TRF2 3'UTR	0.94	0.2242
hsa-mir-135a-2	TRF2 3'UTR	0.94	0.3224
hsa-mir-147b	TRF2 3'UTR	0.94	0.3768
hsa-mir-210	TRF2 3'UTR	0.94	0.1699
hsa-mir-412	TRF2 3'UTR	0.94	0.2723
hsa-mir-451	TRF2 3'UTR	0.94	0.3362
hsa-mir-520h	TRF2 3'UTR	0.94	0.2956
hsa-mir-548e	TRF2 3'UTR	0.94	0.3223
hsa-mir-607	TRF2 3'UTR	0.94	0.3813
hsa-mir-611	TRF2 3'UTR	0.94	0.1003
hsa-mir-647	TRF2 3'UTR	0.94	0.2952
hsa-mir-655	TRF2 3'UTR	0.94	0.0916
hsa-mir-1185-2	TRF2 3'UTR	0.95	0.3687
hsa-mir-122	TRF2 3'UTR	0.95	0.314
hsa-mir-1238	TRF2 3'UTR	0.95	0.2333
hsa-mir-126	TRF2 3'UTR	0.95	0.4083
hsa-mir-1264	TRF2 3'UTR	0.95	0.0956
hsa-mir-1290	TRF2 3'UTR	0.95	0.2729
hsa-mir-1302-5	TRF2 3'UTR	0.95	0.3106
hsa-mir-130b	TRF2 3'UTR	0.95	0.3694
hsa-mir-1323	TRF2 3'UTR	0.95	0.3628
hsa-mir-1910	TRF2 3'UTR	0.95	0.2238
hsa-mir-2052	TRF2 3'UTR	0.95	0.1269
hsa-mir-26b	TRF2 3'UTR	0.95	0.3903
hsa-mir-299	TRF2 3'UTR	0.95	0.3312
hsa-mir-30d	TRF2 3'UTR	0.95	0.0314
hsa-mir-320d-2	TRF2 3'UTR	0.95	0.3482
hsa-mir-449a	TRF2 3'UTR	0.95	0.3013
hsa-mir-559	TRF2 3'UTR	0.95	0.3615
hsa-mir-571	TRF2 3'UTR	0.95	0.3704
hsa-mir-608	TRF2 3'UTR	0.95	0.1601
hsa-mir-620	TRF2 3'UTR	0.95	0.3366
hsa-mir-7-2	TRF2 3'UTR	0.95	0.4091
hsa-mir-1181	TRF2 3'UTR	0.96	0.4154
hsa-mir-1200	TRF2 3'UTR	0.96	0.3817

hsa-mir-1272	TRF2 3'UTR	0.96	0.3463
hsa-mir-153-1	TRF2 3'UTR	0.96	0.403
hsa-mir-15b	TRF2 3'UTR	0.96	0.3732
hsa-mir-187	TRF2 3'UTR	0.96	0.342
hsa-mir-197	TRF2 3'UTR	0.96	0.3016
hsa-mir-20b	TRF2 3'UTR	0.96	0.4258
hsa-mir-373	TRF2 3'UTR	0.96	0.4291
hsa-mir-453	TRF2 3'UTR	0.96	0.3208
hsa-mir-548f-4	TRF2 3'UTR	0.96	0.2877
hsa-mir-552	TRF2 3'UTR	0.96	0.3349
hsa-mir-568	TRF2 3'UTR	0.96	0.339
hsa-mir-1179	TRF2 3'UTR	0.97	0.4067
hsa-mir-1201	TRF2 3'UTR	0.97	0.3887
hsa-mir-1284	TRF2 3'UTR	0.97	0.3329
hsa-mir-1469	TRF2 3'UTR	0.97	0.4456
hsa-mir-183	TRF2 3'UTR	0.97	0.0673
hsa-mir-186	TRF2 3'UTR	0.97	0.4216
hsa-mir-219-1	TRF2 3'UTR	0.97	0.304
hsa-mir-30c-2	TRF2 3'UTR	0.97	0.2267
hsa-mir-548d-2	TRF2 3'UTR	0.97	0.3894
hsa-mir-548f-3	TRF2 3'UTR	0.97	0.39
hsa-mir-548h-4	TRF2 3'UTR	0.97	0.4079
hsa-mir-549	TRF2 3'UTR	0.97	0.1513
hsa-mir-573	TRF2 3'UTR	0.97	0.3729
hsa-mir-603	TRF2 3'UTR	0.97	0.3709
hsa-mir-609	TRF2 3'UTR	0.97	0.4516
hsa-mir-1280	TRF2 3'UTR	0.98	0.4444
hsa-mir-147	TRF2 3'UTR	0.98	0.3961
hsa-mir-29b-1	TRF2 3'UTR	0.98	0.4792
hsa-mir-421	TRF2 3'UTR	0.98	0.3661
hsa-mir-489	TRF2 3'UTR	0.98	0.3882
hsa-mir-586	TRF2 3'UTR	0.98	0.4036
hsa-mir-875	TRF2 3'UTR	0.98	0.4351
hsa-let-7a-2	TRF2 3'UTR	0.99	0.4607
hsa-mir-105-2	TRF2 3'UTR	0.99	0.4613
hsa-mir-1182	TRF2 3'UTR	0.99	0.4694
hsa-mir-1197	TRF2 3'UTR	0.99	0.4737
hsa-mir-1262	TRF2 3'UTR	0.99	0.461
hsa-mir-1270	TRF2 3'UTR	0.99	0.427
hsa-mir-26a-1	TRF2 3'UTR	0.99	0.4492
hsa-mir-342	TRF2 3'UTR	0.99	0.4748
hsa-mir-365-1	TRF2 3'UTR	0.99	0.4196
hsa-mir-369	TRF2 3'UTR	0.99	0.4668
hsa-mir-485	TRF2 3'UTR	0.99	0.4799
hsa-mir-580	TRF2 3'UTR	0.99	0.4863
hsa-mir-587	TRF2 3'UTR	0.99	0.4522
hsa-mir-633	TRF2 3'UTR	0.99	0.4894
hsa-mir-645	TRF2 3'UTR	0.99	0.4731

hsa-mir-92a-2	TRF2 3'UTR	0.99	0.4898
hsa-mir-933	TRF2 3'UTR	0.99	0.4977
hsa-mir-1261	TRF2 3'UTR	1	0.4856
hsa-mir-1288	TRF2 3'UTR	1	0.4795
hsa-mir-149	TRF2 3'UTR	1	0.4829
hsa-mir-196a-2	TRF2 3'UTR	1	0.4731
hsa-mir-208a	TRF2 3'UTR	1	0.4938
hsa-mir-30c-1	TRF2 3'UTR	1	0.4522
hsa-mir-31	TRF2 3'UTR	1	0.4061
hsa-mir-374b	TRF2 3'UTR	1	0.4803
hsa-mir-375	TRF2 3'UTR	1	0.4792
hsa-mir-505	TRF2 3'UTR	1	0.4895
hsa-mir-626	TRF2 3'UTR	1	0.4828
hsa-mir-657	TRF2 3'UTR	1	0.4698
hsa-mir-759	TRF2 3'UTR	1	0.4718
hsa-mir-887	TRF2 3'UTR	1	0.4245
hsa-mir-920	TRF2 3'UTR	1	0.4533
hsa-mir-921	TRF2 3'UTR	1	0.443
hsa-mir-942	TRF2 3'UTR	1	0.3473
hsa-mir-1307	TRF2 3'UTR	1.01	0.475
hsa-mir-188	TRF2 3'UTR	1.01	0.4295
hsa-mir-220b	TRF2 3'UTR	1.01	0.4751
hsa-mir-372	TRF2 3'UTR	1.01	0.3401
hsa-mir-376b	TRF2 3'UTR	1.01	0.4441
hsa-mir-514-3	TRF2 3'UTR	1.01	0.4778
hsa-mir-516a-2	TRF2 3'UTR	1.01	0.4622
hsa-mir-518a-1	TRF2 3'UTR	1.01	0.4688
hsa-mir-557	TRF2 3'UTR	1.01	0.3961
hsa-mir-577	TRF2 3'UTR	1.01	0.4753
hsa-mir-600	TRF2 3'UTR	1.01	0.4293
hsa-mir-602	TRF2 3'UTR	1.01	0.4519
hsa-mir-614	TRF2 3'UTR	1.01	0.4363
hsa-mir-643	TRF2 3'UTR	1.01	0.4444
hsa-mir-1203	TRF2 3'UTR	1.02	0.3583
hsa-mir-129-1	TRF2 3'UTR	1.02	0.2545
hsa-mir-136	TRF2 3'UTR	1.02	0.2914
hsa-mir-145	TRF2 3'UTR	1.02	0.4199
hsa-mir-153-2	TRF2 3'UTR	1.02	0.3226
hsa-mir-509-1	TRF2 3'UTR	1.02	0.4062
hsa-mir-514-2	TRF2 3'UTR	1.02	0.3627
hsa-mir-548a-1	TRF2 3'UTR	1.02	0.0133
hsa-mir-548g	TRF2 3'UTR	1.02	0.4029
hsa-mir-548h-2	TRF2 3'UTR	1.02	0.4194
hsa-mir-569	TRF2 3'UTR	1.02	0.3824
hsa-mir-575	TRF2 3'UTR	1.02	0.428
hsa-mir-660	TRF2 3'UTR	1.02	0.3847
hsa-mir-668	TRF2 3'UTR	1.02	0.3766
hsa-let-7f-2	TRF2 3'UTR	1.03	0.2478

hsa-mir-1253	TRF2 3'UTR	1.03	0.3735
hsa-mir-1283-2	TRF2 3'UTR	1.03	0.4485
hsa-mir-1306	TRF2 3'UTR	1.03	0.3895
hsa-mir-18b	TRF2 3'UTR	1.03	0.3166
hsa-mir-320d-1	TRF2 3'UTR	1.03	0.3483
hsa-mir-361	TRF2 3'UTR	1.03	0.4383
hsa-mir-371	TRF2 3'UTR	1.03	0.3701
hsa-mir-376a-1	TRF2 3'UTR	1.03	0.4261
hsa-mir-454	TRF2 3'UTR	1.03	0.2692
hsa-mir-581	TRF2 3'UTR	1.03	0.3907
hsa-mir-940	TRF2 3'UTR	1.03	0.2777
hsa-mir-181a-2	TRF2 3'UTR	1.04	0.2991
hsa-mir-181b-2	TRF2 3'UTR	1.04	0.2373
hsa-mir-501	TRF2 3'UTR	1.04	0.3421
hsa-mir-556	TRF2 3'UTR	1.04	0.2811
hsa-mir-595	TRF2 3'UTR	1.04	0.2313
hsa-mir-604	TRF2 3'UTR	1.04	0.3613
hsa-mir-770	TRF2 3'UTR	1.04	0.1014
hsa-mir-886	TRF2 3'UTR	1.04	0.245
hsa-let-7g	TRF2 3'UTR	1.05	0.4178
hsa-mir-1251	TRF2 3'UTR	1.05	0.283
hsa-mir-1321	TRF2 3'UTR	1.05	0.4241
hsa-mir-148a	TRF2 3'UTR	1.05	0.3064
hsa-mir-450a-2	TRF2 3'UTR	1.05	0.2371
hsa-mir-579	TRF2 3'UTR	1.05	0.1488
hsa-mir-617	TRF2 3'UTR	1.05	0.3519
hsa-mir-618	TRF2 3'UTR	1.05	0.0863
hsa-mir-625	TRF2 3'UTR	1.05	0.1876
hsa-mir-936	TRF2 3'UTR	1.05	0.1066
hsa-mir-1227	TRF2 3'UTR	1.06	0.1436
hsa-mir-1274a	TRF2 3'UTR	1.06	0.2764
hsa-mir-1304	TRF2 3'UTR	1.06	0.3545
hsa-mir-1322	TRF2 3'UTR	1.06	0.0062
hsa-mir-1825	TRF2 3'UTR	1.06	0.3738
hsa-mir-26a-2	TRF2 3'UTR	1.06	0.3562
hsa-mir-32	TRF2 3'UTR	1.06	0.0545
hsa-mir-320c-2	TRF2 3'UTR	1.06	0.225
hsa-mir-520g	TRF2 3'UTR	1.06	0.1446
hsa-mir-548j	TRF2 3'UTR	1.06	0.0342
hsa-mir-561	TRF2 3'UTR	1.06	0.1173
hsa-mir-574	TRF2 3'UTR	1.06	0.1384
hsa-mir-758	TRF2 3'UTR	1.06	0.2567
hsa-mir-1273	TRF2 3'UTR	1.07	0.3642
hsa-mir-196a-1	TRF2 3'UTR	1.07	0.2008
hsa-mir-203	TRF2 3'UTR	1.07	0.3564
hsa-mir-329-2	TRF2 3'UTR	1.07	0.3732
hsa-mir-497	TRF2 3'UTR	1.07	0.0332
hsa-mir-526a-2	TRF2 3'UTR	1.07	0.1859

hsa-mir-5481	TRF2 3'UTR	1.07	0.1649
hsa-mir-591	TRF2 3'UTR	1.07	0.2501
hsa-mir-605	TRF2 3'UTR	1.07	0.1266
hsa-mir-654	TRF2 3'UTR	1.07	0.2295
hsa-mir-766	TRF2 3'UTR	1.07	0.0071
hsa-mir-941-1	TRF2 3'UTR	1.07	0.2296
hsa-mir-1243	TRF2 3'UTR	1.08	0.267
hsa-mir-1279	TRF2 3'UTR	1.08	0.1306
hsa-mir-1303	TRF2 3'UTR	1.08	0.1148
hsa-mir-1468	TRF2 3'UTR	1.08	0.0865
hsa-mir-146a	TRF2 3'UTR	1.08	0.1236
hsa-mir-20a	TRF2 3'UTR	1.08	0.0493
hsa-mir-30b	TRF2 3'UTR	1.08	0.2434
hsa-mir-548d-1	TRF2 3'UTR	1.08	0.185
hsa-mir-596	TRF2 3'UTR	1.08	0.149
hsa-mir-99b	TRF2 3'UTR	1.08	0.1099
hsa-mir-1249	TRF2 3'UTR	1.09	0.2817
hsa-mir-1255b-1	TRF2 3'UTR	1.09	0.2881
hsa-mir-148b	TRF2 3'UTR	1.09	0.1877
hsa-mir-1977	TRF2 3'UTR	1.09	0.224
hsa-mir-19b-2	TRF2 3'UTR	1.09	0.0347
hsa-mir-302a	TRF2 3'UTR	1.09	0.2502
hsa-mir-452	TRF2 3'UTR	1.09	0.0967
hsa-mir-518e	TRF2 3'UTR	1.09	0.1543
hsa-mir-520f	TRF2 3'UTR	1.09	0.2358
hsa-mir-548b	TRF2 3'UTR	1.09	0.0161
hsa-mir-566	TRF2 3'UTR	1.09	0.2577
hsa-mir-584	TRF2 3'UTR	1.09	0.2299
hsa-mir-1275	TRF2 3'UTR	1.1	0.2596
hsa-mir-1302-7	TRF2 3'UTR	1.1	0.0738
hsa-mir-1305	TRF2 3'UTR	1.1	0.0983
hsa-mir-17	TRF2 3'UTR	1.1	0.2847
hsa-mir-218-2	TRF2 3'UTR	1.1	0.0926
hsa-mir-30e	TRF2 3'UTR	1.1	0.2693
hsa-mir-362	TRF2 3'UTR	1.1	0.3354
hsa-mir-384	TRF2 3'UTR	1.1	0.0614
hsa-mir-511-1	TRF2 3'UTR	1.1	0.2232
hsa-mir-548n	TRF2 3'UTR	1.1	0.1747
hsa-mir-558	TRF2 3'UTR	1.1	0.243
hsa-mir-1294	TRF2 3'UTR	1.11	0.3393
hsa-mir-141	TRF2 3'UTR	1.11	0.2187
hsa-mir-2054	TRF2 3'UTR	1.11	0.2717
hsa-mir-27b	TRF2 3'UTR	1.11	0.0576
hsa-mir-30a	TRF2 3'UTR	1.11	0.1143
hsa-mir-365-2	TRF2 3'UTR	1.11	0.1105
hsa-mir-377	TRF2 3'UTR	1.11	0.1244
hsa-mir-510	TRF2 3'UTR	1.11	0.0295
hsa-mir-516b-1	TRF2 3'UTR	1.11	0.1968

hsa-mir-548a-2	TRF2 3'UTR	1.11	0.1752
hsa-mir-548p	TRF2 3'UTR	1.11	0.2289
hsa-mir-1281	TRF2 3'UTR	1.12	0.1885
hsa-mir-190b	TRF2 3'UTR	1.12	0.1617
hsa-mir-19b-1	TRF2 3'UTR	1.12	0.0275
hsa-mir-494	TRF2 3'UTR	1.12	0.2886
hsa-mir-504	TRF2 3'UTR	1.12	0.2447
hsa-mir-519b	TRF2 3'UTR	1.12	0.0262
hsa-mir-526a-1	TRF2 3'UTR	1.12	0.0252
hsa-mir-548f-1	TRF2 3'UTR	1.12	0.2146
hsa-mir-548h-1	TRF2 3'UTR	1.12	0.197
hsa-mir-106b	TRF2 3'UTR	1.13	0.0673
hsa-mir-517b	TRF2 3'UTR	1.13	0.002
hsa-mir-548i-2	TRF2 3'UTR	1.13	0.1179
hsa-mir-548o	TRF2 3'UTR	1.13	0.2052
hsa-mir-578	TRF2 3'UTR	1.13	0.0467
hsa-mir-1266	TRF2 3'UTR	1.14	0.0746
hsa-mir-320b-2	TRF2 3'UTR	1.14	0.0073
hsa-mir-330	TRF2 3'UTR	1.14	0.2095
hsa-mir-337	TRF2 3'UTR	1.14	0.2164
hsa-mir-367	TRF2 3'UTR	1.14	0.1519
hsa-mir-518a-2	TRF2 3'UTR	1.14	0.1653
hsa-mir-567	TRF2 3'UTR	1.14	0.2003
hsa-mir-627	TRF2 3'UTR	1.14	0.1856
hsa-mir-924	TRF2 3'UTR	1.14	0.1674
hsa-mir-16-2	TRF2 3'UTR	1.15	0.2186
hsa-mir-18a	TRF2 3'UTR	1.15	0.3021
hsa-mir-363	TRF2 3'UTR	1.15	0.0873
hsa-mir-532	TRF2 3'UTR	1.15	0.0541
hsa-mir-588	TRF2 3'UTR	1.15	0.0465
hsa-mir-613	TRF2 3'UTR	1.15	0.1469
hsa-mir-938	TRF2 3'UTR	1.15	0.0775
hsa-mir-1291	TRF2 3'UTR	1.16	0.0225
hsa-mir-484	TRF2 3'UTR	1.16	0.0962
hsa-mir-511-2	TRF2 3'UTR	1.16	0.2248
hsa-mir-590	TRF2 3'UTR	1.16	0.0223
hsa-mir-623	TRF2 3'UTR	1.16	0.0391
hsa-mir-1297	TRF2 3'UTR	1.17	0.1876
hsa-mir-1302-6	TRF2 3'UTR	1.17	0.0792
hsa-mir-765	TRF2 3'UTR	1.17	0.1535
hsa-let-7d	TRF2 3'UTR	1.18	0.113
hsa-mir-216a	TRF2 3'UTR	1.18	0.0847
hsa-mir-218-1	TRF2 3'UTR	1.18	0.09
hsa-mir-339	TRF2 3'UTR	1.18	0.1373
hsa-mir-487b	TRF2 3'UTR	1.18	0.2234
hsa-mir-520c	TRF2 3'UTR	1.18	0.1517
hsa-mir-885	TRF2 3'UTR	1.19	0.0459
hsa-mir-103-2	TRF2 3'UTR	1.2	0.0663

hsa-mir-10a	TRF2 3'UTR	1.2	0.0488
hsa-mir-1202	TRF2 3'UTR	1.2	0.0444
hsa-mir-1913	TRF2 3'UTR	1.21	0.1054
hsa-mir-194-1	TRF2 3'UTR	1.21	0.0636
hsa-mir-320c-1	TRF2 3'UTR	1.21	0.1535
hsa-mir-519c	TRF2 3'UTR	1.21	0.021
hsa-mir-2053	TRF2 3'UTR	1.22	0.0733
hsa-mir-519a-1	TRF2 3'UTR	1.22	0.0293
hsa-mir-100	TRF2 3'UTR	1.23	0.0385
hsa-mir-1180	TRF2 3'UTR	1.23	0.0629
hsa-mir-1228	TRF2 3'UTR	1.23	0.0926
hsa-mir-1292	TRF2 3'UTR	1.23	0.0472
hsa-mir-410	TRF2 3'UTR	1.23	0.0284
hsa-mir-545	TRF2 3'UTR	1.23	0.0522
hsa-mir-652	TRF2 3'UTR	1.23	0.1504
hsa-mir-184	TRF2 3'UTR	1.24	0.0731
hsa-mir-616	TRF2 3'UTR	1.24	0.0468
hsa-mir-892a	TRF2 3'UTR	1.24	0.1116
hsa-mir-1276	TRF2 3'UTR	1.25	0.0067
hsa-mir-215	TRF2 3'UTR	1.25	0.008
hsa-mir-222	TRF2 3'UTR	1.25	0.011
hsa-mir-518d	TRF2 3'UTR	1.25	0.0614
hsa-mir-661	TRF2 3'UTR	1.25	0.1478
hsa-mir-572	TRF2 3'UTR	1.26	0.104
hsa-mir-597	TRF2 3'UTR	1.26	0.1081
hsa-mir-548h-3	TRF2 3'UTR	1.27	0.0558
hsa-mir-943	TRF2 3'UTR	1.27	0.2012
hsa-mir-592	TRF2 3'UTR	1.28	0.015
hsa-mir-323	TRF2 3'UTR	1.29	0.125
hsa-mir-411	TRF2 3'UTR	1.29	0.0047
hsa-mir-329-1	TRF2 3'UTR	1.3	0.0465
hsa-mir-376a-2	TRF2 3'UTR	1.3	0.0529
hsa-mir-381	TRF2 3'UTR	1.31	0.0107
hsa-mir-493	TRF2 3'UTR	1.32	0.0254
hsa-mir-496	TRF2 3'UTR	1.32	0.0199
hsa-mir-520a	TRF2 3'UTR	1.32	0.0442
hsa-mir-520d	TRF2 3'UTR	1.32	0.1326
hsa-mir-10b	TRF2 3'UTR	1.33	0.0248
hsa-mir-302b	TRF2 3'UTR	1.33	0.0344
hsa-mir-34a	TRF2 3'UTR	1.33	0.024
hsa-mir-376c	TRF2 3'UTR	1.33	0.0272
hsa-mir-500	TRF2 3'UTR	1.34	0.1154
hsa-mir-624	TRF2 3'UTR	1.34	0.0835
hsa-mir-1539	TRF2 3'UTR	1.35	0.0182
hsa-mir-449b	TRF2 3'UTR	1.35	0.0337
hsa-mir-1263	TRF2 3'UTR	1.36	0.0898
hsa-mir-34c	TRF2 3'UTR	1.36	0.0133
hsa-mir-302c	TRF2 3'UTR	1.37	0.0537

hsa-mir-455	TRF2 3'UTR	1.37	0.0131
hsa-mir-29c	TRF2 3'UTR	1.38	0.2723
hsa-mir-432	TRF2 3'UTR	1.38	0.0788
hsa-mir-495	TRF2 3'UTR	1.38	0.1284
hsa-mir-502	TRF2 3'UTR	1.38	0.0058
hsa-mir-1260	TRF2 3'UTR	1.39	0.0137
has-mir-328	TRF2 3'UTR	1.41	0.0671
hsa-mir-520b	TRF2 3'UTR	1.41	0.0334
hsa-mir-802	TRF2 3'UTR	1.41	0.0357
hsa-mir-34b	TRF2 3'UTR	1.42	0.0298
hsa-mir-519a-2	TRF2 3'UTR	1.42	0.0341
hsa-mir-518f	TRF2 3'UTR	1.43	0.1042
hsa-mir-216b	TRF2 3'UTR	1.5	0.0253
hsa-mir-1233	TRF2 3'UTR	1.51	0.0378
hsa-mir-518c	TRF2 3'UTR	1.51	0.0633
hsa-mir-190	TRF2 3'UTR	1.52	0.0418
hsa-mir-524	TRF2 3'UTR	1.53	0.0193
hsa-mir-1244	TRF2 3'UTR	1.59	0.037
hsa-mir-1827	TRF2 3'UTR	1.62	0.0336
hsa-mir-220a	TRF2 3'UTR	1.64	0.0206
hsa-mir-450b	TRF2 3'UTR	1.64	0.023
hsa-let-7c	TRF2 3'UTR	1.67	0.0334
has-mir-1301	TRF2 3'UTR	1.77	0.0253
